# Incidence and predictors of serious bleeding during long-term follow-up after acute coronary syndrome in a population-based cohort study

**DOI:** 10.1038/s41598-021-01525-7

**Published:** 2021-11-09

**Authors:** Anna Graipe, Anders Ulvenstam, Anna-Lotta Irevall, Lars Söderström, Thomas Mooe

**Affiliations:** Institution of Public Health and Clinical Medicine, Östersund, Umeå University, Umeå, Sweden

**Keywords:** Stroke, Cardiovascular diseases, Interventional cardiology, Lower gastrointestinal bleeding, Upper gastrointestinal bleeding

## Abstract

Progress in decreasing ischemic complications in acute coronary syndrome (ACS) has come at the expense of increased bleeding risk. We estimated the long-term, post-discharge incidence of serious bleeding, characterized bleeding type, and identified predictors of bleeding and its impact on mortality in an unselected cohort of patients with ACS. In this population-based study, we included 1379 patients identified with an ACS, 2010–2014. Serious bleeding was defined as intracranial hemorrhage (ICH), bleeding requiring hospital admission, or bleeding requiring transfusion or surgery. During a median 4.6-year follow-up, 85 patients had ≥ 1 serious bleed (cumulative incidence, 8.6%; 95% confidence interval (CI) 8.3–8.9). A subgroup of 557 patients, aged ≥ 75 years had a higher incidence (13.4%) than younger patients (6.0%). The most common bleeding site was gastrointestinal (51%), followed by ICH (27%). Sixteen percent had a recurrence. Risk factors for serious bleeding were age ≥ 75 years, lower baseline hemoglobin (Hb) value, previous hypertension or heart failure. Serious bleeding was associated with increased mortality. Bleeding after ACS was fairly frequent and the most common bleeding site was gastrointestinal. Older age, lower baseline Hb value, hypertension and heart failure predicted bleeding. Bleeding did independently predict mortality.

## Introduction

Bleeding complications following acute coronary syndrome (ACS) have attracted considerable attention in recent years. The increased intensity in antithrombotic treatment during the last decades to improve outcomes in ACS has decreased risk for ischemic events but at the expense of greater bleeding risk^1–3^. Bleeding is associated with both morbidity and mortality^4–6^. Previous studies have found that bleeding after ACS has an association with mortality similar to that of myocardial infarction^6^, and major bleeding correlates with a more prolonged mortality risk compared with ischemic events^7^. The incidence of post-discharge major bleeding within 1 year in patients with ACS has been reported as 1.3–5.6% in previous randomized trials (RCTs) and observational studies^8–10^, and up to one-third of patients discharged on dual antiplatelet therapy (DAPT) are reported to have had a bleeding complication within 12 months post-discharge^8^. Comparison of bleeding incidences among studies is difficult because of differences in the studied populations, follow-up times, in-hospital management, antithrombotic drugs used, and bleeding definitions^11,12^. Overall, bleeding incidence has been higher in observational studies than in RCTs because of older populations with more comorbidities and risk factors in the former^13^.

DAPT, consisting of aspirin plus a P2Y12 receptor antagonist, is the cornerstone of treatment in patients with ACS*.* An invasive strategy with percutaneous coronary intervention (PCI) requires dual antithrombotic therapy for up to 1 year to minimize the risk of stent thrombosis and new ischemic events, although the time may be shorter or longer depending on the type of stent and the patient’s bleeding risk^14^. In an observational study, medically (non-invasively) treated patients were reported to have a higher risk for re-hospitalization related to bleeding, as well as an increased mortality risk compared to invasively treated patients^15^. Previously identified predictors of bleeding include advanced age, prior ischemic or hemorrhagic stroke, previous bleeding, hypertension, renal failure, female sex, lower weight, diabetes, atrial fibrillation, and a low hemoglobin (Hb) value^16–20^. The most common type of major bleeding is gastrointestinal (GI), and the most fatal is intracranial^21^. Most previous studies report in-hospital bleeding, and few studies have reported follow-up beyond 1 year. There is a lack of long-term follow-up observational studies of an unselected ACS population, and few studies report recurrent bleeding events.

The aims of this study were to estimate the long-term, post-discharge incidence of bleeding, characterize the type of bleeding, and identify predictors of bleeding and its impact on mortality in an unselected cohort of ACS patients.

## Material and methods

In this population-based study, we included all patients who were identified with an ACS during the inclusion period of the Nurse-Based Age-Independent Intervention to Limit Evolution of Disease After Acute Coronary Syndrome (NAILED-ACS) Risk Factor trial. Briefly, the study cohort consisted of all patients admitted to Östersund Hospital with ACS from January 1, 2010, to December 31, 2014. Östersund Hospital is the only hospital in Jämtland-Härjedalen County, a geographically large, rural area with approximately 126,000 inhabitants. To identify all patients who were admitted to the hospital with an ACS diagnosis, medical records were reviewed for all patients with suspected ACS on a daily basis. ACS was defined as unstable angina (UA), consisting of chest pain and ischemic changes on an electrocardiogram, or acute myocardial infarction (AMI) type 1, according to the universal definition of myocardial infarction^22^.

### Data collection and adjudication of endpoints

Patients in the NAILED-ACS cohort were followed from the day of discharge until death, a move out of the county, or December 31, 2017. Serious bleeds were identified through review of the discharge records for all hospitalizations at the Department of Internal Medicine. To capture all bleeding complications regardless of hospital department, we identified bleeding diagnosis (Table S1) in the local hospital inpatient register, which covers all hospital admissions, and validated the diagnosis by a search of the medical records. When a patient had more than one serious bleed, up to the first three were included. Identification and review of potential endpoint events were performed by three medical doctors, all members of the study team. The review process followed a standardized workflow routine, and events were strictly evaluated according to study outcome definitions. Each reviewer worked with their assigned cases independently, but consecutive meetings were held to reach consensus in complicated cases.

Serious bleeding was defined as an intracranial hemorrhage (ICH), bleeding that required hospital admission, or bleeding that required transfusion or surgery. All bleeds were subclassified as intracranial (epidural, subdural, intracerebral hematoma, subarachnoid bleed), GI (upper, lower, or non-classified), and other serious bleeding (intraocular, retroperitoneal, or urinary tract bleeding)*.* The classification was made according to International Statistical Classification of Diseases and Related Health Problems (ICD-10) codes (Table S1).

Clinical baseline characteristics and medications at discharge were extracted from the NAILED-ACS database. The variable “smoking” was defined as smoking during the past month. The variable “atrial fibrillation” was defined as previous atrial fibrillation or atrial fibrillation during hospitalization. The variable “estimated glomerular filtration rate” (eGFR) was calculated using the Chronic Kidney Disease Epidemiology Collaboration equation (the CKD-EPI)^23^, and the limit for decreased kidney function was defined as ≤ 60 mL/min/1.72 m^2^. The limit for obesity was set to a body mass index ≥ 30 kg/m^2^. The value of systolic blood pressure was taken from the day before discharge and thus after the acute stage. In Sweden, elementary school lasts 9 years; to study if education level affected bleeding outcome, we divided education level by elementary school level vs. higher. Lipid treatment at discharge was a statin in 85% of the patients. Intervention during the hospitalization was either PCI or coronary artery bypass graft (CABG). Heart failure during hospitalization was defined as heart failure on X-ray, the presence of pulmonary rales, or having had intravenous treatment with diuretics.

### Statistical analysis

Baseline data are presented as means for continuous variables and as counts and percentages for categorical variables. Data for patients with and without a bleeding event were compared with Student’s independent samples t-tests for continuous variables, and with the Pearson Chi-square test for categorical variables. Age is presented as medians with 25th and 75th percentiles and was compared using the Mann–Whitney U test.

Kaplan − Meier analysis was used to estimate the cumulative incidence of bleeding in the whole cohort and stratified by age (cutoff, 75 years). Kaplan–Meier analysis was also used to describe mortality in patients with and without a bleeding event, and comparisons between groups were performed using the log-rank test.

A multivariable Cox proportional hazards regression model was used to identify predictors of bleeding. Variables included in the univariable analysis had previously been described as risk factors or were of potential importance. All variables with a *p* value < 0.5 were then included in the multivariable Cox model. Non-significant variables were excluded stepwise according to their level of significance during subsequent runs until reaching only significant predictors. The assumption of proportional hazard was verified using scaled Schoenfeld residuals. Because death might occur before bleeding, a competing risk analysis also was performed according to Fine–Gray, using the same stepwise approach. In addition, a multivariable Cox regression analysis with post discharge bleeding as a time-dependent variable was used to identify predictors of death. Results are presented as hazard ratios (HRs) with 95% confidence intervals (CIs).

Statistical analyses were performed using SPSS (version 25.0; IBM Corp, Armonk, NY, USA) and SAS software (version 9.4; SAS Institute Inc, Cary, NC, USA).

### Ethics

The Regional Ethics Committee in Umeå approved this study on October 28, 2009 (Dnr: 09-142M), with supplements on June 10, 2013 (Dnr: 2013-204-32M) and January 13, 2015 (Dnr: 2014-416-32 M). The study was conducted in accordance with relevant guidelines and regulations. All participants signed an informed consent prior to randomization.

## Results

### Participants

**Figure 1:**
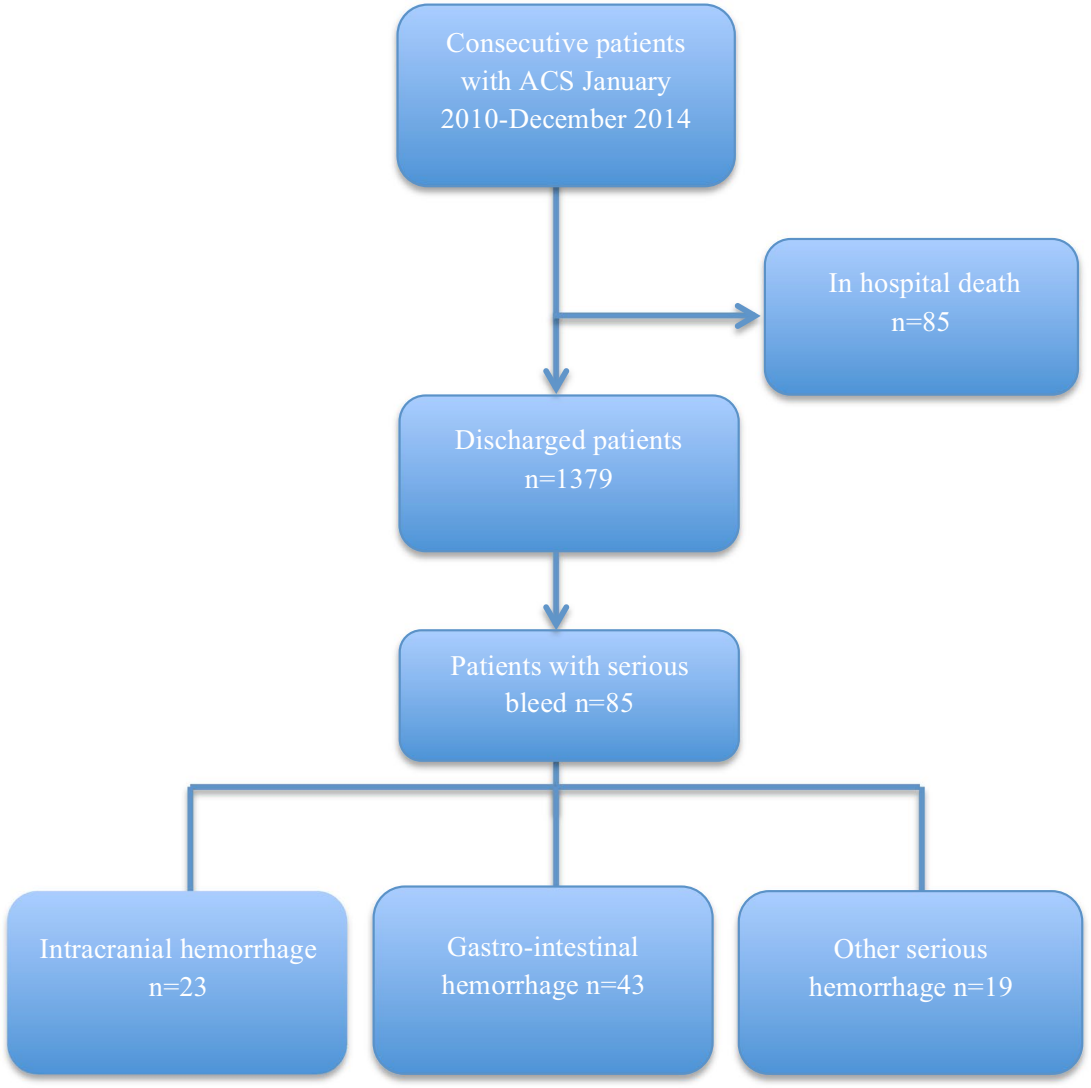
Study flow chart.

A total of 1379 patients with ACS were followed from discharge and up to 8 years. A study flowchart is presented in Fig. 1, and baseline characteristics are shown in Table 1. The mean age of participants was 72 years, 35.5% were female, and 57.4% had only a basic level of education (i.e., elementary school or similar). Non-ST-elevation myocardial infarction (NSTEMI) was the most common index event, occurring in 63.5% (n = 876), followed by ST-elevation myocardial infarction (STEMI) in 28.4% (n = 392) and UA in 8.0% (n = 110). Slightly more than half (53.9%) of the population underwent invasive revascularization therapy (PCI/CABG), and a fifth (19.3%) were treated with thrombolysis. In the whole cohort, 74% (n = 1021) were treated with dual antithrombotic therapy with aspirin and a P2Y12 inhibitor (i.e., DAPT), and 1.7% (n = 24) patients had triple antithrombotic therapy (DAPT and an oral anticoagulant (OAC)).

**Table 1:** Baseline characteristics stratified by bleeding.

Patient characteristics	All patients n = 1379	Bleed n = 85	Non-bleed n = 1294	*p*
Age, median (quartiles)	72 (63.81)	77 (69.83)	71 (63.81)	< 0.001
Women, n (%)	489 (35.5)	29 (34.1)	460 (35.6)	0.785
BMI kg/m^2^ (mean)	27.0	27.0	27.1	0.155
Former/current smoker, n (%)	874 (60.7)	54 (63.5)	784 (61.0)	0.645
Basic education, n (%)	736 (57.4)	51 (65.4)	685 (56.8)	0.139
Systolic BP (mean)	132	132	132	0.827
Baseline Hb, mean g/L	140	134	141	< 0.001
B-glucose (mean)	6.9	7.8	6.9	0.031
eGFR (mean)	76.3	68.5	76.8	< 0.001
**ACS diagnosis n (%)**				
STEMI	392 (28.4)	27 (31.8)	365 (28.2)	0.484
NSTEMI	876 (63.6)	50 (58.8)	826 (63.9)	0.348
UA	110 (8.0)	8 (9.4)	102 (7.9)	0.616
**Revascularization, n (%)**				
PCI	642 (43.9)	30 (35.3)	606 (46.9)	0.038
CABG	147 (10.0)	6 (7.1)	134 (10.4)	0.329
Thrombolysis	266 (19.3)	16 (18.8)	250 (19.3)	0.908
**Comorbidities, n (%)**				
Previous angina	319 (23.2)	29 (34.1)	290 (22.5)	0.01
Previous AMI	290 (21.0)	24 (28.2)	266 (20.6)	0.093
Previous PCI	120 (8.7)	12 (14.1)	107 (8.3)	0.068
Previous CABG	120 (8.7)	13 (15.3)	107 (8.3)	0.026
Previous ischemic stroke/TIA	111 (8.1)	6 (7.1)	105 (8.1)	0.299
Previous hemorrhagic stroke	7 (0.5)	1 (1.2)	6 (0.5)	0.371
Previous PAD	35 (2.5)	4 (4.7)	31 (2.4)	0.190
Hypertension	797 (57.8)	62 (72.9)	735 (56.8)	0.004
Diabetes	299 (21.7)	23 (27.1)	276 (21.3)	0.216
Atrial fibrillation	232 (16.8)	19 (22.4)	213 (16.5)	0.161
Congestive heart failure	70 (5.1)	10 (11.8)	60 (4.6)	0.004
COPD	75 (5.4)	8 (9.4)	67 (5.2)	0.096
**Baseline medication at discharge, n (%)**				
Lipid-lowering treatment	1200 (87.1)	71 (83.5)	1129 (87.3)	0.313
Beta-blocker	1213 (88.0)	73 (85.9)	1140 (88.2)	0.530
ACE inhibitor/ARB	1053 (76.4)	67 (78.8)	986 (76.3)	0.589
Aspirin	1277 (92.7)	74 (87.1)	1203 (93.0)	0.040
P2Y12 receptor inhibitor	1083 (78.6)	63 (74.1)	1020 (78.9)	0.299
Anticoagulant	114 (8.3)	13 (15.3)	101 (7.8)	0.015

*n* number of patients, *BMI* body mass index, *systolic BP* blood pressure at discharge, *eGFR* estimated glomerular filtration rate calculated using mL/min/1.73 m^2^, *ACS* acute coronary syndrome, *STEMI* ST-elevation myocardial infarction, *NSTEMI* non-ST-elevation myocardial infarction, *UA* unstable angina, *PCI* percutaneous coronary intervention, *CABG* coronary artery bypass graft, *TIA* transient ischemic attack, *PAD* peripheral artery disease, *ACE* angiotensin-converting enzyme, *ARB* angiotensin receptor blocker.

### Bleeding incidence and subtype of bleeding

**Figure 2:**
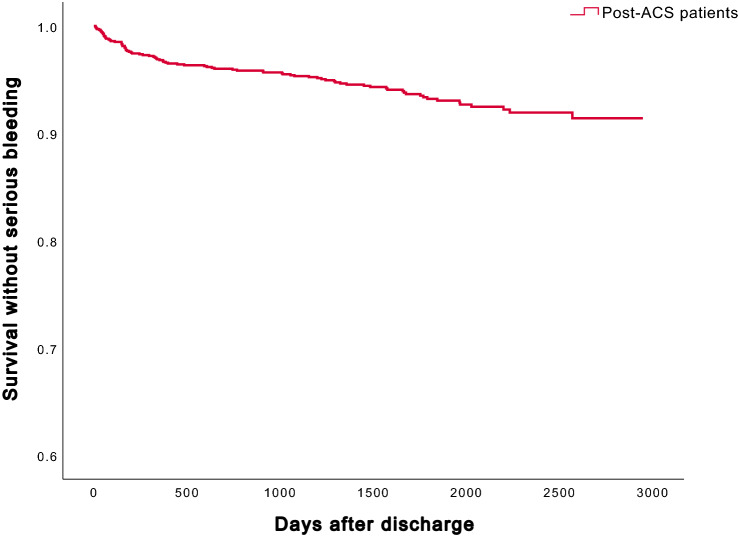
Cumulative incidences without serious bleedings during long-term follow-up.

**Figure 3:**
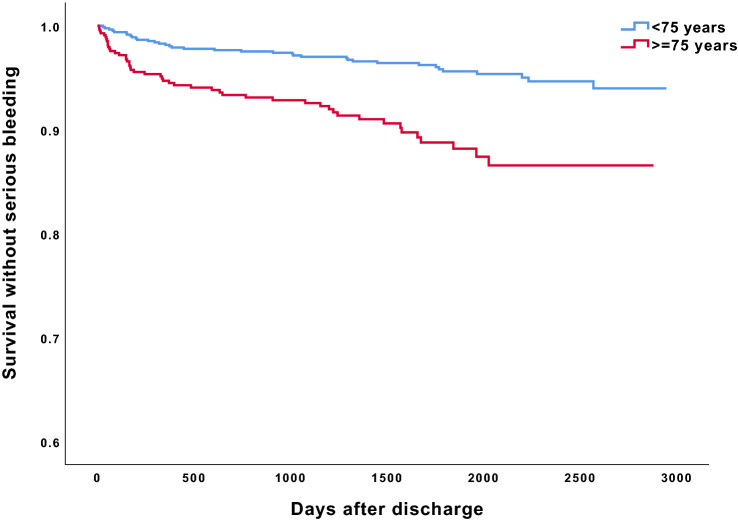
Cumulative incidences without serious bleedings during long-term follow-up, stratified by age.

During a median follow-up of 4.6 years, 85 patients had at least one serious bleeding event, for a cumulative incidence of 8.6% (95% confidence interval (CI) 8.3–8.9%) (Fig. 2). The 1-year incidence was 3.0% (95% CI 2.7–3.3%), or 42 patients. In the subgroup analysis of patients aged ≥ 75 years (n = 557), the long-term cumulative incidence of serious bleeding was significantly higher at 13.4% (95% CI 12.9–14.0%) compared with patients aged < 75 years, at 6.0% (95% CI 5.9–6.2%; *p* < 0.001) (Fig. 3).

The most common bleeding type was GI, occurring in 51% (n = 43) of events, followed by ICH in 27% (n = 23). Of the 23 patients with an ICH, five had an intracerebral hematoma and nine had a subdural or a subarachnoid hemorrhage, respectively. Fourteen patients had more than one bleeding episode post-discharge, and the second bleeding was of GI origin in 79% (n = 11/14). Three patients had a third bleeding, all of GI origin (Table 2). In patients with more than one bleed, the median age was higher than overall, at 79 (interquartile range 68–89) years.

**Table 2:** Fourteen patients with more than one bleeding episode, with bleeding localization presented in order of events.

Age	First bleed	Second bleed	Third bleed
67	Other	Upper GI	
67	Upper GI	Upper GI	Upper GI
68	SAH	Lower GI	
75	SAH	Subdural hematoma	
79	Other	Unspecified GI	
78	SAH	Unspecified GI	
78	SAH	Upper GI	
88	Other	Upper GI	Lower GI
88	Upper GI	Upper GI	Upper GI
91	Unspecified GI	Unspecified GI	
68	Upper GI	Upper GI	
94	Other	Other	
83	Lower GI	Upper GI	
92	Upper GI	Other	

*GI* gastrointestinal, *SAH* subarachnoid hemorrhage, *other* includes intraocular, retroperitoneal, and urinary tract bleeding.

### Predictors of bleeding

Patients who had a bleeding event during follow-up were older, and several comorbid conditions were more prevalent in this group compared with patients without a bleeding event (Table 1). Comorbidities included a history of hypertension, congestive heart failure, angina, previous CABG, lower mean baseline Hb, and a lower mean eGFR. At discharge, treatment with warfarin was more common among those who later experienced bleeding, whereas aspirin treatment was more common among patients without a bleeding event. The use of P2Y12 did not differ between groups, and there was no significant difference in proportions being treated with DAPT (67.1% with a bleed vs. 74.6% without a bleed). No patients discharged with triple antithrombotic therapy had a bleeding event during follow-up.

Results of the univariable Cox regression analysis are shown in the supplementary material (Table S2). In the multivariable Cox analysis (Table 3), significant predictors of bleeding were age ≥ 75 years (HR 2.0; 95% CI 1.2–3.1), previous hypertension (HR 1.8; 95% CI 1.1–2.9), and previous heart failure (HR 2.2; 95% CI 1.1–4.5). An increase in baseline Hb (HR 0.97; 95% CI 0.96–0.99) and female sex was associated with decreased risk (HR 0.6; 95% CI 0.4–0.97). Of note, bleeding risk did not differ according to level of education, subtype of index event (STEMI/NSTEMI/UA), invasive revascularization, or pharmacologic treatment at discharge. Analyses of interactions between all variables using competing risk analysis gave the same significant results as the multivariable Cox analysis.

**Table 3:** Multivariable Cox regression analysis of predictors for bleeding post-discharge after ACS.

Predictor	HR	CI	*p*
Age ≥ 75 years	2.0	1.2–3.1	0.005
Female sex	0.6	0.4–0.97	0.039
Hypertension	1.8	1.1–2.9	0.02
Previous heart failure	2.2	1.1–4.5	0.002
Baseline Hb, per g/L increase	0.97	0.96–0.99	0.0002

*HR* hazard ratio, *CI* confidence interval, *ACS* acute coronary syndrome.

### Mortality

**Figure 4:**
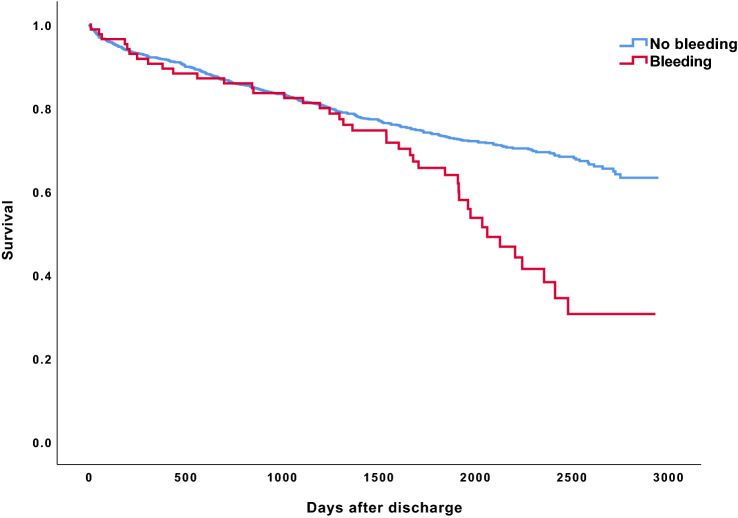
Cumulative survival after ACS, stratified by bleeding.

A total of 404 patients died during follow-up, with a significantly higher mortality among those who had a bleeding event (48.2%) vs. those who did not (28.1%; *p* < 0.001)*.* As illustrated in Fig. 4, the between-group difference in mortality appeared after 3–3.5 years (*p* < 0.001). In the time-dependent multivariable Cox analysis (Supplement Table S3) with adjustment for age and comorbid conditions, having a bleeding episode was associated with increased risk of death (HR 1.6; 95% CI 1.1–2.4). Also, high age and several of the comorbid conditions were significantly associated with mortality. Higher BMI, higher education level and a history of diabetes were associated with decreased mortality. Treatment with statins and intervention during hospitalization were associated with decreased risk and treatment with P2Y12 were associated with increased risk (HR1.4; 95% CI 1.05–1.7).

## Discussion

In this population-based cohort study of 1379 patients with ACS, the cumulative incidence of serious bleeding complications post-discharge was 8.6% during a median follow-up of 4.6 years and 3.0% within 1 year. In patients aged ≥ 75 years, the long-term incidence (13.4%) was more than twice that among patients age < 75 years (6.0%). The most common bleeding type was GI, with predominantly upper GI hemorrhages, followed by ICH. Older age, lower baseline Hb, hypertension, and previous heart failure predicted serious bleeding, female sex was associated with lower bleeding risk. A post discharge bleeding did independently predict mortality.

### Incidence and type of bleeding

We carried out a meticulous follow-up of bleeding events requiring hospital care, and to our knowledge, no other study has such a long follow-up of unselected ACS patients. One study had a similar follow-up time (4.9 years), but was restricted to STEMI patients and showed an incidence of GI bleedings of 7.8% from the time of PCI (i.e., admission)^20^. Of note, these authors reported a 30-day incidence of 3.9%, which is considerably higher than in our cohort. This difference may reflect a high in-hospital bleeding risk and adjacent to hospital discharge, associated with antithrombotic treatments and procedure-related bleeding. Our more diverse population and capture only of post-discharge events may have contributed to the divergence. Another study of patients treated with PCI and drug-eluting stents (62% stable angina) with a follow-up of 7–8 years showed a 5-year cumulative incidence of major bleeding (Bleeding Academic Research Consortium 3–5) of 7.4% and 8-year incidence of 10.8%. The 5-year incidence was slightly higher than ours (6.8% at 5 years) even though those authors used a definition including more severe bleedings and most of the patients were in stable condition^24^. However, the continuation of DAPT beyond one year was relatively common. Voss et al., with a follow-up time of approximately 2 years, found a post-discharge incidence of 7.4% for bleeding-related re-hospitalization. Bleeding events were identified through a diagnosis registry using ICD codes similar to ours. In contrast, the diagnoses were not validated against the medical records for confirmation, which may have led to an overestimation of clinically relevant events because registry-based diagnoses often include a broader definition and secondary diagnoses^21^.

Most clinical trials with “long term” follow-up have a 1-year follow-up duration for bleeding risk. In the Clopidogrel in Unstable Angina to Prevent Recurrent Events (CURE) trial, the 1-year incidence of CURE major bleeding was 3.7% in patients treated with clopidogrel vs. 2.7% with placebo^3^. In the Trial to Assess Improvement in Therapeutic Outcomes by Optimizing Platelet Inhibition with Prasugrel–Thrombolysis in Myocardial Infarction (TRITON–TIMI 38), the 15-month incidence of Thrombolysis in Myocardial Infarction (TIMI) major bleeding was 2.4% in prasugrel-treated patients vs. 1.8% in clopidogrel-treated patients^1^. In the Platelet Inhibition and Patient Outcomes (PLATO) trial, the one-year incidence of TIMI major bleeding was 2.8% with ticagrelor vs. 2.2% with clopidogrel^2^. In comparison, our one-year cumulative incidence was 3.0%. However, major bleeding is a broad concept, and differences in bleeding definitions make comparison difficult.

ICH is more strictly defined and therefore more easily compared across different studies. In our study, the cumulative incidence of ICH within 1 year was 0.7%, which is higher than in the above-mentioned clinical trials. In the CURE trial, which reported hemorrhagic stroke, the incidence was 0.1% and equal between the clopidogrel and placebo groups. In TRITON, the incidence of ICH was 0.3% (equal between the prasugrel and clopidogrel groups), and in PLATO, it was 0.34% with ticagrelor vs. 0.19% with clopidogrel. Our higher incidence of ICH may be explained by higher age and more comorbidity compared to these RCTs. Also, we included all types of intracranial bleedings, even those of traumatic origin.

Compared with previous observational studies with similar bleeding definitions and cohorts, our results are consistent^10,15^ or the values are lower^25,26^. Brinkert et al. found a post-discharge 1-year bleeding rate of 2.6%, and the bleeding rate was significantly lower in patients treated with PCI. Patients treated medically only are often older with more comorbidity, which can explain the differences between the groups. Gray et al. found a 1-year incidence of serious bleeding of 3.2% in the total cohort, but among patients older than 75 years, the bleeding rate was higher at 5.6%, in line with our findings. These results show the strong impact of age on bleeding risk^10^. Patients with more than one bleeding episode in our study had a mean age even higher than patients with one bleeding episode. Results from the Swedish Web-system for Enhancement and Development of Evidence-based care in Heart disease Evaluated According to Recommended Therapies (SWEDEHEART) registry, with a bleeding definition and patient characteristics similar to ours, showed a 1-year (post-discharge) bleeding incidence of 4.0–4.8% during 2010–2015. Overestimation of diagnoses obtained from the National Patient Registry may explain the difference (i.e., there was no review of the medical records for confirmation)^25^.

STEMI patients have previously been reported to have a lower incidence of major bleeding compared to NSTEMI patients^25,27^, a difference related to a lower mean age and a lower risk profile overall among STEMI patients. In contrast to these previous reports, we found no difference in bleeding incidence between STEMI and NSTEMI patients. It should be noted, however, that the total number of bleeding events was relatively small in our study, making all subgroup analyses less conclusive. Also, the difference in age between STEMI and NSTEMI patients was less pronounced in our cohort.

Consistent with other studies, the gut^15,21,28^ was the most frequent identifiable source of bleeding in our study. It was also the most common location for a second bleeding event, regardless of the location of the first site. Recurrence of any type of bleeding seems fairly common, as high as 26% within the first 12 month of hospital discharge^29^. A previous study found a more than doubled risk of GI bleeding after a first occurrence^20^. The use of proton pump inhibitors has diminished the risk of GI bleeding in patients treated with antithrombotics and is especially important in elderly patients^30,31^. GI bleeding is relatively common and leads to disability and discontinuation of drugs, increasing the risk of ischemic events. There is a fine balance between timing of antithrombotic treatment withdrawal for bleeding and the risk of a new ischemic event, and new guidelines recommend early resumption of antithrombotics^32^.

### Predictors

We could confirm some of the previously recognized predictors of bleeding: age, lower baseline Hb, previous hypertension, and previous heart failure^16,29,33^. We could not confirm kidney failure or previous stroke/transient ischemic attack to be associated with increased bleeding risk^34,35^. In previous studies, kidney failure has predicted bleeding after ACS (HRs 1.3–1.9)^35,36^, and in a study of patients with kidney failure (not post-ACS), bleeding risk significantly increased in correlation with decreased eGFR^37^. In our study, however, kidney function was relatively good in both groups, which may explain the divergent result, and we would have needed a larger study population to capture any impact of mild kidney failure.

A previous stroke (ischemic, and especially hemorrhagic) has been previously identified as an important predictor of ICH^36,38^ but does not necessarily predict any bleeding event. We had relatively few ICH events and few patients with a previous hemorrhagic stroke. These factors may partly explain why we could not confirm a previous cerebrovascular event as a predictor in our cohort.

Female sex was associated with decreased risk of post-discharge bleeding in our study cohort, in line with the PLATO trial where female sex was associated with an overall lower risk for spontaneous (non-procedure) major bleed^9,39^. Other studies have found diverging results regarding an association between female sex and bleeding risk post-discharge^29,40–42^. Women seem to have more minimal and nuisance bleeding complications post-discharge compared to men^16,40^, small bleedings that was not included in our cohort study. The lower risk in women could be by chance and because of unknown confounders such as less adherence to antithrombotics among women^40,43^.

### Mortality

We found increased mortality among patients with a bleeding event in the long term, and having a bleeding was a significant predictor for all-cause death, as reported in several other studies^4,5,44^. Mortality was equal between patients with and without a bleeding event during the first 3 years post-discharge but then increased in patients with a bleeding event. Surprisingly, higher BMI (≥ 30 kg/m^2^) and diabetes were protective and treatment with P2Y12 was associated with increased risk of death. Residual confounding may explain these findings but we cannot rule out a negative impact of a more aggressive platelet inhibition in the long run^45^. Furthermore, the structured follow-up of diabetic subjects in Swedish health care may be of importance for their long-term prognosis^46^.

## Conclusion

With a median follow-up of 4.6 years in a population-based ACS cohort, the cumulative incidence of re-hospitalization for serious bleeding was 8.6%. Patients aged ≥ 75 years had a more than doubled incidence of serious bleeding compared with patients < 75 years. GI bleeding was most common, and recurrence of bleeding was relatively frequent. Higher age, lower baseline Hb, prior hypertension, and previous heart failure were associated with bleeding. Female sex was associated with decreased bleeding risk. Patients with bleeding had a higher mortality and a post discharge bleeding was a significant predictor of increased mortality. To decrease bleeding risk after ACS, the future challenge is to identify the vulnerable patient, individualize treatment, and repeatedly reevaluate bleeding risk during follow-up.

### Strengths and limitations

Our cohort is relatively small, and our study is observational, with the inherent related limitations. Nonetheless, the data were carefully collected with full coverage of all ACS in the catchment area. We performed an accurate adjudication of bleeding endpoints requiring hospitalization, and follow-up covered a long time period. We cannot rule out that patients experienced bleeding complications that were not documented in patient records or that was handled in primary care, but because we included only serious events, this seems unlikely. We used a pragmatic bleeding definition that did not require laboratory values, which is not directly comparable to standard bleeding definitions in randomized trials. The definition we used has been used in previous observational studies^10,15,26^.

We had no information about previous bleeding except for previous ICH, and we did not know if the patients had continued or changed treatment with antithrombotic drugs post-discharge. The only OAC included was warfarin, so the effect of the newer OACs needs further study.

## Supplementary Information

Supplementary Tables.

## References

[1] Wiviott SD (2007). Prasugrel versus clopidogrel in patients with acute coronary syndromes. N. Engl. J. Med..

[2] Wallentin L (2009). Ticagrelor versus clopidogrel in patients with acute coronary syndromes. N. Engl. J. Med..

[3] Yusuf S (2001). Effects of clopidogrel in addition to aspirin in patients with acute coronary syndromes without ST-segment elevation. N. Engl. J. Med..

[4] Eikelboom JW (2006). Adverse impact of bleeding on prognosis in patients with acute coronary syndromes. Circulation.

[5] Rao SV (2005). Impact of bleeding severity on clinical outcomes among patients with acute coronary syndromes. Am. J. Cardiol..

[6] Ducrocq G (2017). Balancing the risk of spontaneous ischemic and major bleeding events in acute coronary syndromes. Am. Heart J..

[7] Mehran R (2009). Associations of major bleeding and myocardial infarction with the incidence and timing of mortality in patients presenting with non-ST-elevation acute coronary syndromes: A risk model from the ACUITY trial. Eur. Heart J..

[8] Ismail N (2019). Incidence and prognostic impact of post discharge bleeding post acute coronary syndrome within an outpatient setting: A systematic review. BMJ Open.

[9] Becker RC (2011). Bleeding complications with the P2Y12 receptor antagonists clopidogrel and ticagrelor in the PLATelet inhibition and patient outcomes (PLATO) trial. Eur. Heart J..

[10] Garay A (2018). Prediction of post-discharge bleeding in elderly patients with acute coronary syndromes: Insights from the BleeMACS Registry. Thromb. Haemost..

[11] Steinhubl SR, Kastrati A, Berger PB (2007). Variation in the definitions of bleeding in clinical trials of patients with acute coronary syndromes and undergoing percutaneous coronary interventions and its impact on the apparent safety of antithrombotic drugs. Am. Heart J..

[12] Mehran R (2011). Standardized bleeding definitions for cardiovascular clinical trials: A consensus report from the Bleeding Academic Research Consortium. Circulation.

[13] Mortensen J (2008). Incidence of bleeding in 'real-life' acute coronary syndrome patients treated with antithrombotic therapy. Cardiology.

[14] Valgimigli M (2018). 2017 ESC focused update on dual antiplatelet therapy in coronary artery disease developed in collaboration with EACTS: The Task Force for dual antiplatelet therapy in coronary artery disease of the European Society of Cardiology (ESC) and of the European Association for Cardio-Thoracic Surgery (EACTS). Eur. Heart J..

[15] Brinkert M (2017). Incidence and prognostic implications of late bleeding after myocardial infarction or unstable angina according to treatment strategy. Can. J. Cardiol..

[16] Moscucci M (2003). Predictors of major bleeding in acute coronary syndromes: The global registry of acute coronary events (GRACE). Eur. Heart J..

[17] Mehran R (2010). A risk score to predict bleeding in patients with acute coronary syndromes. J. Am. Coll. Cardiol..

[18] Simonsson M (2019). Development and validation of a novel risk score for in-hospital major bleeding in acute myocardial infarction: The SWEDEHEART Score. J. Am. Heart Assoc..

[19] Alfredsson J (2017). Predicting the risk of bleeding during dual antiplatelet therapy after acute coronary syndromes. Heart (British Cardiac Society).

[20] Kikkert WJ (2015). Predictors and prognostic consequence of gastrointestinal bleeding in patients with ST-segment elevation myocardial infarction. Int. J. Cardiol..

[21] Voss WB, Lee M, Devlin GP, Kerr AJ (2016). Incidence and type of bleeding complications early and late after acute coronary syndrome admission in a New Zealand cohort (ANZACS-QI-7). N. Z. Med. J..

[22] Thygesen K (2007). Universal definition of myocardial infarction. Circulation.

[23] Levey AS (2009). A new equation to estimate glomerular filtration rate. Ann. Intern. Med..

[24] Miura K (2019). Long-term incidence and details of bleeding events after everolimus-eluting stent implantation—7-8-year outcomes. Circ. J..

[25] Simonsson M (2020). Temporal trends in bleeding events in acute myocardial infarction: Insights from the SWEDEHEART registry. Eur. Heart J..

[26] Sorensen R (2009). Risk of bleeding in patients with acute myocardial infarction treated with different combinations of aspirin, clopidogrel, and vitamin K antagonists in Denmark: A retrospective analysis of nationwide registry data. Lancet.

[27] Bacquelin R (2016). Safety of prasugrel in real-world patients with ST-segment elevation myocardial infarction: 1-year results from a prospective observational study (Bleeding and Myocardial Infarction Study). Arch. Cardiovasc. Dis..

[28] Chen Y (2019). A risk score to predict postdischarge bleeding among acute coronary syndrome patients undergoing percutaneous coronary intervention: BRIC-ACS study. Catheter. Cardiovasc. Interv..

[29] Ismail N (2019). Bleeding after hospital discharge following acute coronary syndrome: Incidence, types, timing, and predictors. J. Am. Heart Assoc..

[30] Li L, Geraghty OC, Mehta Z, Rothwell PM (2017). Age-specific risks, severity, time course, and outcome of bleeding on long-term antiplatelet treatment after vascular events: A population-based cohort study. Lancet.

[31] Mo C (2015). Proton pump inhibitors in prevention of low-dose aspirin-associated upper gastrointestinal injuries. World J. Gastroenterol..

[32] Halvorsen S (2017). Management of antithrombotic therapy after bleeding in patients with coronary artery disease and/or atrial fibrillation: expert consensus paper of the European Society of Cardiology Working Group on Thrombosis. Eur. Heart J..

[33] Subherwal S (2009). Baseline risk of major bleeding in non-ST-segment-elevation myocardial infarction: The CRUSADE (Can Rapid risk stratification of Unstable angina patients Suppress ADverse outcomes with early implementation of the ACC/AHA Guidelines) bleeding score. Circulation.

[34] Ducrocq G (2013). A history of stroke/transient ischemic attack indicates high risks of cardiovascular event and hemorrhagic stroke in patients with coronary artery disease. Circulation.

[35] Raposeiras-Roubin S (2018). Development and external validation of a post-discharge bleeding risk score in patients with acute coronary syndrome: The BleeMACS score. Int. J. Cardiol..

[36] Graipe A, Binsell-Gerdin E, Soderstrom L, Mooe T (2015). Incidence, time trends, and predictors of intracranial hemorrhage during long-term follow-up after acute myocardial infarction. J. Am. Heart Assoc..

[37] Molnar AO (2016). The risk of major hemorrhage with CKD. J. Am. Soc. Nephrol..

[38] Raposeiras-Roubín S (2019). Incidence, predictors and prognostic impact of intracranial bleeding within the first year after an acute coronary syndrome in patients treated with percutaneous coronary intervention. Eur. Heart J. Acute Cardiovasc. Care.

[39] Husted S (2014). The efficacy of ticagrelor is maintained in women with acute coronary syndromes participating in the prospective, randomized, PLATelet inhibition and patient outcomes (PLATO) trial. Eur. Heart J..

[40] Holm A (2018). Bleeding complications after myocardial infarction in a real world population—An observational retrospective study with a sex perspective. Thromb. Res..

[41] Grodecki K (2018). Gender-related differences in post-discharge bleeding among patients with acute coronary syndrome on dual antiplatelet therapy: A BleeMACS sub-study. Thromb. Res..

[42] Hess CN (2014). Sex-based differences in outcomes after percutaneous coronary intervention for acute myocardial infarction: A report from TRANSLATE-ACS. J. Am. Heart Assoc..

[43] Mehran R (2013). Cessation of dual antiplatelet treatment and cardiac events after percutaneous coronary intervention (PARIS): 2 year results from a prospective observational study. Lancet.

[44] Ducrocq G (2015). Association of spontaneous and procedure-related bleeds with short- and long-term mortality after acute coronary syndromes: An analysis from the PLATO trial. EuroIntervention.

[45] Szummer K (2020). Comparison between ticagrelor and clopidogrel in elderly patients with an acute coronary syndrome: Insights from the SWEDEHEART Registry. Circulation.

[46] The Swedish National Diabetes Register (NDR) annual report 2020. www.ndr.nu.

