# Supplementary material for: Incidence and predictors of serious bleeding during long-term follow-up after acute coronary syndrome in a population-based cohort study
Source: Sci Rep. 2021 Nov 9;11:21967. doi: 10.1038/s41598-021-01525-7 (PMC8578330; doi:10.1038/s41598-021-01525-7)
Supplement: Supplementary file 1 — Supplementary Tables. [file 41598_2021_1525_MOESM1_ESM.docx]

**Incidence and Predictors of Serious Bleeding during Long-term Follow-up after Acute Coronary Syndrome in a Population-based Cohort Study**

Anna Graipe MD, PhD^1^; Anders Ulvenstam MD, PhD^1^; Anna-Lotta Irevall MD, PhD^1^; Lars Söderström, MSc^1^; Thomas Mooe, Senior Professor^1^

1. Institution of Public Health and Clinical Medicine, Östersund, Umeå University, Umeå, Sweden

**Supplement tables**

Table S1. ICD codes for serious bleeding diagnosis

- D 62.9 – Acute post-hemorrhagic anemia
- D 50.0 – Iron-deficiency anemia secondary to blood loss
- H 11.3 – Conjunctival hemorrhage
- H 31.3 – Choroidal hemorrhage
- H 35.6 – Retinal hemorrhage
- H 43.1, H 45.0 – Vitreous hemorrhage
- H 92.2 – Hemorrhage from the ear
- I 60 – Subarachnoid hemorrhage
- I 61 – Intracerebral hemorrhage
- I 62 – Non-traumatic intracranial hemorrhage
- I 69, I 69.1, I 69.2 – Sequel of intracranial hemorrhage
- I 84.1, I 84.4, I 84.8 – Hemorrhoids
- I 85.0, I 98.3 – Esophageal varices
- K 22.6 – Gastro-esophageal laceration hemorrhage
- K 25-28 – Acute peptic ulcer
- K 29 – Acute hemorrhagic gastritis
- K 62.5 – Hemorrhage of anus and rectum
- K 92.0 – Hematemesis
- K 92.1 – Melena
- K 92.2 – Gastrointestinal hemorrhage, unspecified
- M 25.0 – Hemarthrosis
- N 42.1 – Congestion and hemorrhage of prostate
- N 93.8, N 93.9 – Abnormal urine and vaginal bleeding
- N 95.0 – Postmenopausal hemorrhage
- R 04.1, R 04.2, R 04.8, R 04.9 – Hemorrhage from throat and airways
- R 31.9 – Hematuria
- S 06.4, S 06.5, S 06.6 – Epidural hemorrhage, traumatic subarachnoid and subdural
- R 58.9 – Unspecified hemorrhage
- T 81.0 – Hemorrhage as complication of surgery

| Predictor | HR (95% CI) | *p* |
| --- | --- | --- |
| Age | 1.1 (1.0–1.1) | <0.001 |
| Age ≥75 | 2.7 (1.8–4.2) | <0.001 |
| Female sex | 1.0 (0.7–1.6) | 0.94 |
| BMI ≥30 kg/m^2^ | 0.7 (0.4–1.2) | 0.23 |
| Baseline Hb, per g/L increase | 0.97 (0.96-0.98) | <0.001 |
| B-glucose | 1.0 (1.0–1.1) | 0.03 |
| Systolic blood pressure at discharge | 1.0 (1.0–1.0) | 0.67 |
| Previous or current smoker | 1.0 (0.7–1.6) | 0.85 |
| Education level | 0.6 (0.4–1.0) | 0.06 |
| Previous hypertension | 2.2 (1.3–3.5) | 0.002 |
| Previous COPD | 2.2 (1.1–4.6) | 0.03 |
| Previous diabetes | 1.4 (0.9–2.3) | 0.13 |
| Previous or new atrial fibrillation | 1.7 (1.0–2.8) | 0.04 |
| Kidney failure (eGFR <60 ml/min/1.73 m^2^) | 1.9 (1.2–3.0) | 0.01 |
| Previous heart failure | 3.7 (1.9–7.2) | <0.001 |
| Previous ischemic stroke/TIA | 1.0 (0.5–2.4) | 0.95 |
| Previous intracranial hemorrhage | 3.6 (0.5–26.5) | 0.20 |
| Previous MI | 1.7 (1.1–2.8) | 0.02 |
| Previous PAD | 2.3 (0.9–6.3) | 0.10 |
| Previous PCI | 1.7 (0.9–3.2) | 0.08 |
| Previous CABG | 2.3 (1.3–4.2) | 0.01 |
| STEMI | 1.1 (0.7–1.8) | 0.60 |
| PCI during hospitalization | 0.6 (0.4–0.9) | 0.01 |
| Thrombolysis during hospitalization | 0.9 (0.5–1.6) | 0.71 |
| Heart failure during hospitalization | 1.9 (1.2–3.1) | 0.01 |
| ACEI/ARB at discharge | 1.1 (0.7–1.8) | 0.76 |
| Lipid treatment at discharge | 0.6 (0.3–0.9) | 0.02 |
| Beta-blockers at discharge | 0.8 (0.4–1.4) | 0.38 |
| Aspirin at discharge | 0.5 (0.2–0.8) | 0.01 |
| P2Y12 receptor antagonist at discharge | 1.3 (0.8–2.1) | 0.28 |
| Oral anticoagulant at discharge | 2.3 (1.3–4.2 | 0.01 |
| Intervention during hospitalization | 0.5 (0.3–0.7) | <0.001 |

Table S2. Predictors of post-discharge bleeding in an unadjusted Cox regression model.

HR, hazard ratio; CI, confidence interval; BMI, body mass index; education level, more than 9 years of elementary school; COPD, chronic obstructive pulmonary disease; eGFR, estimated glomerular filtration rate; STEMI, ST-elevation myocardial infarction; TIA, transient ischemic attack; MI, myocardial infarction; PAD, peripheral artery disease; PCI, percutaneous coronary intervention; CABG, coronary artery bypass graft; ACEI, angiotensin-converting enzyme inhibitor; ARB, angiotensin receptor blocker; intervention during hospitalization, patients treated with either PCI or CABG.

| Predictor | HR (95% CI) | *p* |
| --- | --- | --- |
| Age ≥75 | 2.6 (1.9–3.4) | <0.001 |
| Female sex | 0.9 (0.7–1.1) | 0.19 |
| BMI ≥30 kg/m^2^ | 0.7 (0.5–0.9) | 0.005 |
| Systolic blood pressure at discharge | 0.99 (0.99–1.0) | 0.004 |
| Education level | 0.7 (0.5–0.9) | 0.003 |
| Diabetes | 0.7 (0.6–0.9) | 0.003 |
| Previous or new atrial fibrillation | 1.4 (1.1–1.8) | 0.01 |
| Kidney failure (eGFR<60 ml/min/1.73 m^2^) | 1.5 (1.2–1.9) | 0.0003 |
| Previous congestive heart failure | 1.5 (1.1–2.1) | 0.02 |
| Previous MI | 1.5 (1.2–1.9) | 0.0005 |
| Previous CABG | 1.5 (1.1–2.0) | 0.02 |
| Lipid treatment at discharge | 0.4 (0.3–0.6) | <0.001 |
| Intervention during hospitalization | 0.4 (0.3–0.6) | <0.001 |
| STEMI | 0.7 (0.6-0.96) | 0.03 |
| P2Y12 inhibitor | 1.4 (1.05-1.8) | 0.02 |
| Heart failure during hospitalization | 1.3 (1.03-1.7) | 0.03 |
| Post discharge serious bleeding | 1.6 (1.1-2.4) | 0.009 |

Table S3. Predictors of post-discharge mortality after ACS in a multivariable Cox regression model with post discharge bleeding as a time-dependent variable.

HR, hazard ratio; CI, confidence interval; BMI, body mass index; education level more than 9 years of elementary school; eGFR, estimated glomerular filtration rate; MI, myocardial infarction; CABG, coronary artery bypass graft; intervention during hospitalization, either percutaneous coronary intervention (PCI) or CABG ; STEMI, ST elevation myocardial infarction.
